# Dose-Response Aligned Circuits in Signaling Systems

**DOI:** 10.1371/journal.pone.0034727

**Published:** 2012-04-05

**Authors:** Long Yan, Qi Ouyang, Hongli Wang

**Affiliations:** 1 State key Laboratory for Mesoscopic Physics and School of Physics, Peking University, Beijing, China; 2 Center for Theoretical Biology, Peking University, Beijing, China; 3 The Peking-Tsinghua Center for Life Sciences at School of Physics, Beijing, China; University of Maribor, Slovenia

## Abstract

Cells use biological signal transduction pathways to respond to environmental stimuli and the behavior of many cell types depends on precise sensing and transmission of external information. A notable property of signal transduction that was characterized in the *Saccharomyces cerevisiae* yeast cell and many mammalian cells is the alignment of dose-response curves. It was found that the dose response of the receptor matches closely the dose responses of the downstream. This dose-response alignment (DoRA) renders equal sensitivities and concordant responses in different parts of signaling system and guarantees a faithful information transmission. The experimental observations raise interesting questions about the nature of the information transmission through DoRA signaling networks and design principles of signaling systems with this function. Here, we performed an exhaustive computational analysis on network architectures that underlie the DoRA function in simple regulatory networks composed of two and three enzymes. The minimal circuits capable of DoRA were examined with Michaelis-Menten kinetics. Several motifs that are essential for the dynamical function of DoRA were identified. Systematic analysis of the topology space of robust DoRA circuits revealed that, rather than fine-tuning the network's parameters, the function is primarily realized by enzymatic regulations on the controlled node that are constrained in limiting regions of saturation or linearity.

## Introduction

**Figure 1:**
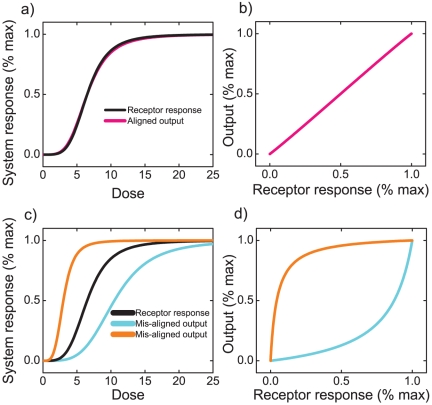
Schematic illustration of dose-response alignment and misalignment. (a) Example of aligned dose response of the receptor and the pathway output (such as gene expressions), in which the receptor and the downstream output response to signal in coordination and with essentially equal sensitivity. (b) The transfer function generated from (a) is essentially linear, and the difference in the receptor occupancy is evenly distinguishable in the pathway output. (c) Examples of misaligned dose responses, in which the response sensitivity of the receptor is very different from that of the downstream response. The pathway output would be saturated while there are still spare receptors (red), or the vice versa (blue), the receptor response is saturated in advance of downstream response. (d) Nonlinear relationships between the receptor occupancy and downstream output generated from (c), making either the receptor response or the downstream output less distinguishable.

Cells use signal transduction pathways to respond to environmental stimuli. Receptors on cell surface sense the signal, trigger subsequent intracellular signaling cascades, and eventually create a change either in the activity of enzymes in the cytoplasm or in gene expressions in the nucleus. The behavior of many cell types depends on precise sensing and transmission of environmental conditions. In a class of cellular signaling systems, experimental studies of the input-output properties demonstrate that the systems show a notable feature named dose-response alignment (DoRA): the dose-response curve of receptor occupancy aligns closely with dose-response curves of downstream responses [1] (Figure 1a). Evidences for such DoRA property were previously demonstrated in many mammalian cell signaling systems. Experiments on mitogenic responses of human and mouse fibroblast cells to the stimulation of epidermal growth factor found a linear relationship between the receptor occupancy and mitogenic response [2]. Dose-response aligned signaling systems observed in earlier years include the insulin [3], acetylcholine [4], thyroid stimulating hormone [5], and angiotensin II [6]. The most extensively investigated signal transduction pathway that bears the feature of DoRA is the mating pathway of *Saccharomyces cerevisiae* yeast cells. Extensive DoRAs were observed from the upstream to the downstream of the pathway: the receptor occupancy is aligned with the G-protein activation/dissociation, the accumulated amount of pheromone-activated Ste12, pheromone-inducible gene expressions, and the cell-cycle arrest, despite that there are many intermediate signaling events in the system [1], [7]–[9].

DoRA is defined by matched dose-response curves or a linear relationship between receptor occupancy and downstream responses (Figure 1a, 1b). As reported in Ref. [1], DoRA guarantees that the entire range of receptor occupancies corresponds evenly to the entire range of downstream responses; and any changes in the receptor occupancy can be evenly discriminated in downstream outputs. By contrast, in a misaligned signaling system (Figure 1c), the downstream responses would be saturated while there are apparent “spare” receptors, or the vice versa, the receptor would be saturated ahead of the downstream with underutilized response capacity. In both cases, the receptor occupancy is no long a constant proportion to the downstream response (Figure 1d), and the extracellular information will not be effectively transmitted and read out in the downstream outputs. The DoRA property exhibited in many cellular signaling systems presents a device in which the output at different measurement points mirrors the percentage of receptor occupancy. This guarantees that the external ligand concentration can be relayed precisely deeper into the cell on which the cell operates to make decision. The DoRA feature indicates a function of close match of information processing in signaling systems, which is important for the fidelity of information transmission.

The DoRA property should be preferential in signaling systems where the precision of sensing and transmission of the external signal of ligand concentration is required for proper cellular functions. In budding yeast, the ability of precise discrimination between high- and low- concentration pheromone-secreting partners is important for the mating behavior. Experimental studies showed that the ability of discrimination relies on the optimal transmission of information about the pheromone concentration [1], [10]. Both distinguishable receptor occupancy and distinguishable downstream response of the pathway are necessary. The study of mating projection orientation in spatial gradients of pheromone with engineered hypersensitive yeasts (deprived of the DoRA feature) showed that hypersensitive cells do not orient their mating projections as precisely as wild type cells [11]. While the DoRA property is advantageous for precise sensing and transmission of external stimulus, signaling systems with saturated or switch-like responses without the DoRA property are plenty [12]–[16]. The non-DoRA feature might be preferred in signaling systems where the sensitivity to the external signal of ligand concentration is more important than the sensing precision of the signal. In fact, model and experimental studies showed that in relays of signal transduction with multistep biochemical reactions, the more normal behaviors are switch-like or hypersensitive response to stimulus: the dose-response curve at downstream steps is not aligned but become progressively more sensitive [14]–[16]. It moves to the left at each downstream step and is steepened. The system response thus becomes switch-like which can be triggered at low amounts of environmental stimulus [15], [16]. As it is unusual that the DoRA property is preserved through chains of biochemical reactions, some control mechanisms should operate in precise signal transduction processes [1], [14].

The experimental findings of DoRA in budding yeast and many mammalian cell signaling systems raise interesting questions about the nature of the information transmission and design principles underlying the notable function in signaling systems. For the frequently observed characteristic in signaling networks, we ask theoretically, what type signal processing and mechanisms will allow the system's downstream responses align with the receptor response. For this purpose, we restricted ourselves to enzymatic interaction networks and applied the method that was used to investigate biochemical adaption by Ma et al [17] to address the question. We enumerated all possible two-node and three-node networks by imposing the constraint of linear relationship between the responses of input and output nodes to screen out the circuits capable of DoRA. The mechanisms for simple and core circuits to achieve DoRA were resolved on the base of Michaelis-Menten kinetics. Several simple motifs that are essential for the function of DoRA were identified. Systematic analysis of the topology of DoRA circuits suggests that there are mainly two ways to achieve DoRA. The function is primarily realized by enzymatic regulations of either the output node or the intermediate node that are constrained in saturated or linear regions instead of fine-tuning any one of the parameters.

## Results

### Full-space screening for DoRA circuits

**Figure 2:**
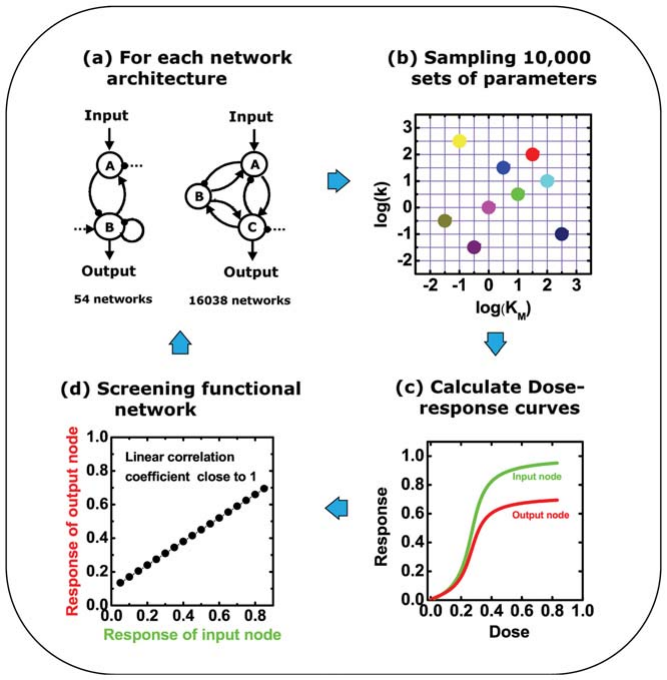
Screening for networks with DoRA function. For each network with two nodes and three nodes, 10,000 random sets of parameters were assigned. The corresponding kinetic equations were solved numerically to obtain the dose-response curves. Linear correlation coefficients were calculated subsequently from the relationship between the responses of the output node and the input node. The number of parameter sets that render good linear output-input dependence (Q-value) measures the ability for the corresponding network to achieve dose-response alignment.

To identify simple network topologies that can achieve the function of DoRA, we first carry out an extensive screening for dose-response aligned circuits in all regulatory networks that are composed of two and three interacting enzymes (Figure 2a). In each network, enzyme *A* receives upstream input (dose), and enzyme *B* (in two-node networks) or enzyme C (in three-node networks) transmits outputs (response). Each node in the network is assumed to have a fixed total concentration (normalized to 1). An enzyme can be in active or inactive form and can be transformed into each other. The transformation is assumed to be catalyzed by activated enzymes in the network or by a basal enzyme in the background. A link is either positive or negative. For instance, a positive link from node *A* to node *B* implies that the active form of enzyme *A* is able to transform enzyme *B* from its inactive form to active form. We assume that the inter-conversion between active and inactive forms of enzyme is reversible. If a node has no agonistic regulations, a background constitutive enzyme is assumed to perform the opposed regulation (such as the dashed positive and negative arrows in Figure 2a). The kinetic response of the network is described by Michaelis-Menten rate equations, which are characterized by the Michaelis-Menten constants (*K_M_*'s) and catalytic rate constants (*k*'s) of enzymes.

**Figure 3:**
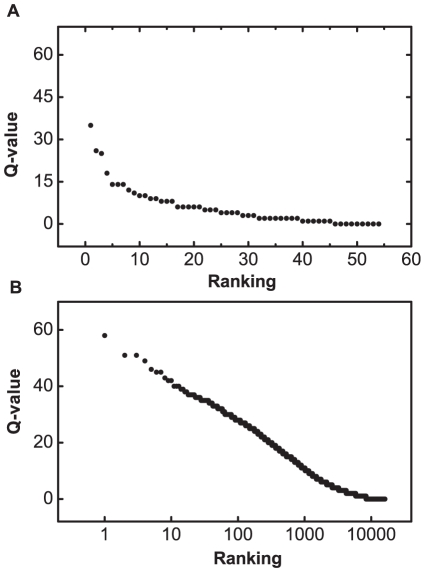
Ranking of the Q-values. Q-values for all two-node networks (A) and all three networks (B), both showing exponential-like dependence on the ranking.

For each network topology, 10,000 sets of circuit parameters are sampled in the parameter space (Figure 2b) [17], [18]. The dose-responses of input and output nodes are obtained numerically by checking stationary steady state solutions of Michaelis-Menten equations (Figure 2c). The resulting behavior of an individual network with each parameter set is characterized by a linear correlation coefficient for the dose-responses of output- and input-node upon stimuli (Figure 2d). The behavior with a coefficient close to 1 is considered to be capable of DoRA. For each particular circuit architecture, we focus on how many parameter sets can achieve DoRA. The larger is the number of parameter sets (defined as Q-value) the more robust the circuit is considered to perform the function. Figure 3 demonstrates the ranking of Q-values for all the networks we have considered. Both two- and three-node networks are drastically different in their ability to achieve DoRA function. The Q-value falls exponentially versus the ranking, showing that only a small fraction of networks is robust for the DoRA function. Most of the 54 possible two-node networks are capable of DoRA with 4 networks having Q-values larger than 15, and 9 networks having zero Q-value. In 16038 possible three-node networks, there are 633 possible topologies with Q-values larger than 15; and only about 0.025% of all 1.6×10^8^ possible topology/parameter sets are found to be capable of DoRA function.

**Figure 4:**
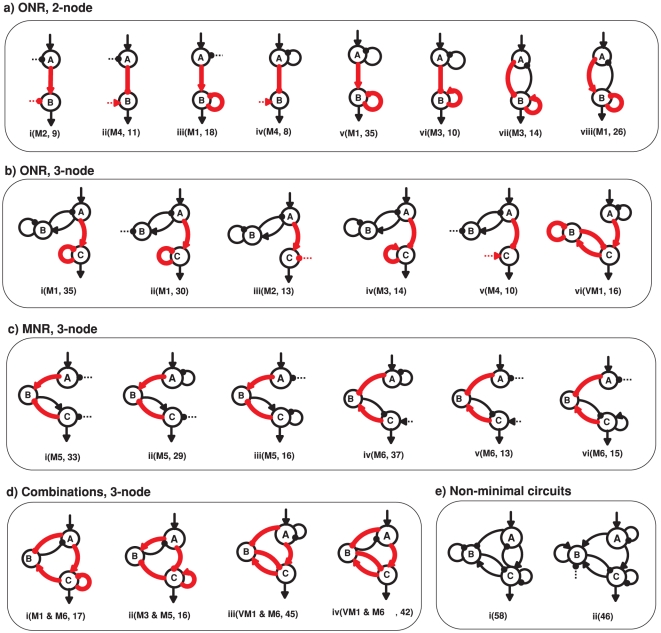
Simplest functional networks. The minimal networks that were identified to have the DoRA function (a–d). The red links in a network are regulations that are confined to saturation or linear regions, i.e., motifs (denoted by M_i_) that are listed in Figure 5. The red links in b(vi) is a variation of M1 motif (VM1). The number in the bracket is Q-value for the circuit. All two-node minimal networks were found to achieve DoRA by constraining the enzyme regulation of the output node (a, ONR). Three-node simplest networks can achieve DoRA by constrained regulations of either the output node A (b, ONR) or the middle buffering node B (c, MNR) or both (d, ONR/MNR). Two examples of non-minimal functional networks are illustrated in (e).

Figure 4(a–d) demonstrates the simplest functional networks obtained computationally. When cooperated with regulations from background enzymes (dashed links), DoRA can be achieved in simplest two-node networks having a single A-to-B link (Figure 4a(i, ii)). In these simplest functional networks, the roles of the basal regulations that make enzyme *A* and enzyme *B* reversible can be taken over by node *A* and node *B* that feedback on themselves (Figure 4a(iii, iv, v, vi)). The basal repression on node *A* can also be replaced by a negative link from node *B* (Figure 4a(vii, viii)). As each node is agonistically regulated, the minimal number of links in these simplest two-node functional networks is three (including possible dashed links from basal enzymes) (Figure 4a(i–viii)). Functional networks that are more complex are possible when additional links are appropriately appended to the core topologies.

For simplest networks composed of three nodes, the minimal number of links is five (include dashed links from background enzymes). According to distinct features in architecture, the minimal topologies that have DoRA function could be primarily classified into different categories (Figure 4b, 4c). The first category is mainly featured by a direct regulation of output node *C* by input node *A* (Figure 4b), i.e., the external stimulus is transmitted directly from the input node to the output node. In comparison with the minimal networks in Figure 4a, the regulation patterns of the output node in three-node networks are primarily identical to those in two-node networks. In this category, the DoRA function is mainly involved with the regulation on node *C* by node *A*. In the second category of simplest three-node circuits with DoRA function (Figure 4c), the information is transmitted indirectly from input-node *A* to output-node *C* through the intermediate node *B* that is regulated in opposition by node *A* and node *C*. As will be discussed later in detail, the intermediate node *B* in this category plays a central role in achieving a linear relationship between node *A* and node *C*. Functional networks that are more complex are observed to have *A-B-C* loops (Figure 4d) as hybridizations of the above two categories of minimal circuits.

### Mechanisms for achieving DoRA

#### Output node regulation (ONR)

Based on the minimal architecture that are sufficient for DoRA, we next check the functional networks by analyzing specific examples to clarify the underlying mechanisms. For the simplest two-node network with only a link from node *A* to output node *B* (Figure 4a(i)), the kinetic equations read,
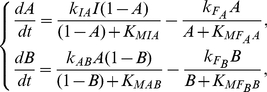
(1)where *A* and *B* represent the concentrations of active forms of enzymes *A* and *B*. The concentrations of basal enzymes that oppose the activations of nodes *A* and *B* have been taken into the rate coefficients 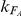 and 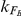 (hereinafter the same). Examination of the parameter sets that enable the circuit to achieve DoRA indicates that the two Michaelis-Menten constants 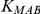 and 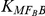 tend to be constrained: 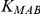 is much smaller than the inactive form of enzyme *B* and 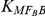 is much larger than the active form of enzyme *B*. This indicates that the activation of enzyme *B* by enzyme *A* approaches saturation and the deactivation of enzyme *B* by the basal enzyme works in the linear region. The condition of saturation (or linearity) is that the substrate concentration is much higher (or lower) than the corresponding Michaelis-Menten constant, i.e., 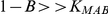 (or 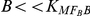). The equation for active form of enzyme *B* is thus approximated as,
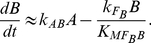
(2)The steady state solution is,
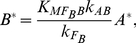
(3)which is independent of the input strength *I*. The output *B* can transiently respond to changes in the input, but at stable steady state the linear relationship between node *A* and node *B* is fixed, which is the condition for DoRA.

**Figure 5:**
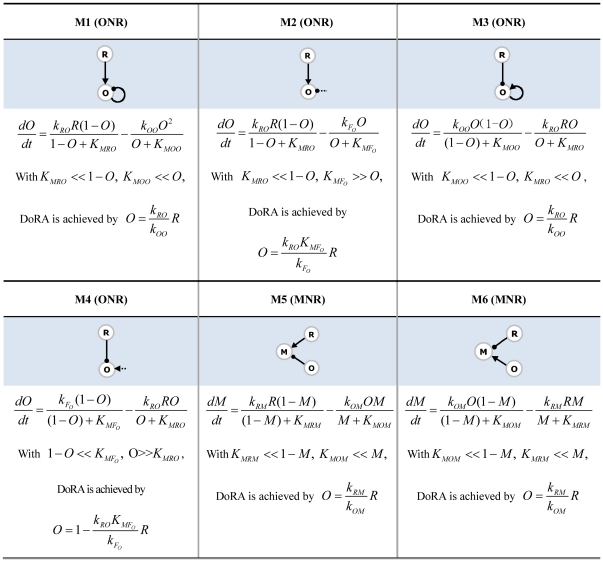
Motifs and mechanisms that are sufficient for achieving DoRA function. The nodes labeled with “R”, “O”, and “M” are input node, output node, and middle buffering node, respectively. Dashed links are assumed as regulations from basal enzymes in the environment.

For other minimal networks composed of two-node in Figure 4a, the Michaelis-Menten constants involved in regulations on the output *B* are all found to be similarly constrained, i.e., the reactions occur primarily either in the saturated or in the linear regions. We summarized the motifs and the underlying mechanisms to achieve DoRA for two-node minimal networks in Figure 5 (M1–M4). Based on the motifs, more complex networks are possible when additional links are properly imposed. The mechanism that gives rise to DoRA in two-node networks has two general features: the output node is regulated directly by the input node, and the corresponding enzymatic reactions are constrained in limiting regions.

We next consider functional minimal circuits composed of three nodes. As can be seen in Figure 4b, the above mechanisms for DoRA in two-node networks are shared by the three-node networks in which the input node *A* regulates directly the output node *C*. In this category, only the equation for the output node *C* is involved with both the input and output nodes, and the regulation on the output node is key for establishing a linear response dependence of node *C* on node *A*. Node *B* plays an assistant role that merely regulates the behavior of node *A* in cooperation with the input. For the instance of network in Figure 4b(i), the regulation pattern of the output node is identical to the two-node circuits in Figure 4a (iii, v, and viii) with the common DoRA motif M1.

The circuits discussed above share the commonality that the input node regulates directly the output node, and the kinetic equation for the output node is explicitly dependent on both the input node and output node. The regulations on the output node are consequently crucial for the DoRA function, which were constrained in limiting regions of saturation or linearity. We classify the circuits that achieve DoRA function with this mechanism as output-node-regulation (ONR) type (M1–M4 in Figure 5). As depicted in Figure 4a and 4b(i–v), all of the simplest networks of two-node and a fraction of simplest three-node networks capable of DoRA belong to this type. The circuit Figure 4b(vi) is a variation of M1 where the negative self-loop of the output node *C* is replaced with a negative feedback buffered by node *B*. In this variational M1 mechanism, the regulations on both node *B* and node *C* need to be constrained to saturation.

#### Middle node regulation (MNR)

The second category of minimal functional networks of three nodes in Figure 4c feature a different character in architecture from those in Figure 4b: the regulation of node *A* on node *C* is absent (possible link from node *C* to node *A*) and node *B* is regulated by nodes *A* and *C* with opposing signs. In these circuits, only the output node *B* is involved with both input and output nodes, and is the pivot in achieving the DoRA function. For an example, the kinetic equations for the circuit in Figure 4c(i) read,
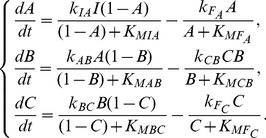
(4)The linear relationship between *A* ^*^ and *C* ^*^ would be most readily established through 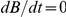. When the opposed regulations of node *A* and node *C* on node *B* are constrained to saturation, i.e., 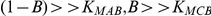, the steady state solutions of *A* and *C* achieves DoRA through,
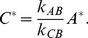
(5)Generally, the regulations on the intermediate node *B* in this category are constrained and node *B* plays a central role in the realization of DoRA function. We classify this type of functional networks that achieve DoRA by constrained regulations on the buffering node *B* as middle-node-regulation (MNR) type. The regulation pattern on the node *B* in the network Figure 4c(i), i.e., node *B* is activated by node *A* and repressed by node *C*, is shared by the networks of Figure 4c(ii, iii). Two primary motifs for the MNR type DoRA are summarized in Figure 5 (M5, M6). In both cases, the alignment is fulfilled through saturated regulations on the buffering node *B*. It would be noted that for the MNR type circuits in which node *C* is partially regulated by a basal enzyme, there exists an additional scenario for DoRA. For the network in Figure 4c(i) described by Eq. (4), one has 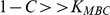 and 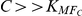 when the regulations on node *C* work in saturation regions, leading to a constant steady state
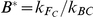
independent of the input level *I*. This results in the linear relationship 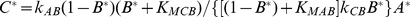 from the steady state solution of node *B* in Eq. (4). In these particular MNR type circuits (e.g., Figure 4c(i,ii,iv,v)), the additional way to DoRA is realized by controlling the level of active enzyme *B* at a constant by constraining the regulations of output node *C* to saturation.

#### Combinational type

The ONR and MNR mechanisms for DoRA discussed above are not exclusive to each other. They can coexist to form combinational ONR-MNR functional circuits. Figure 4d depicts several such hybridized minimal circuits. In this combinational category, either the regulations of node *B* or node *C* or both would be constrained in limiting regions in order to achieve DoRA function. For the functional network of Figure 4d(i), the kinetic equations read,
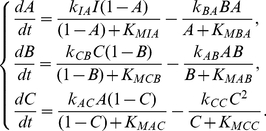
(6)In contrast to the kinetics for circuits in Figure 4b and 4c, both the equations for node *B* and node *C* are explicitly dependent on variables *A* and *C*. According to Figure 5, constrained regulations on either node *B* (M6) or node *C* (M1) and on both nodes could all lead to DoRA function. In the final case, the regulations on both nodes should coordinate in order to achieve a consistent linear relationship between steady states *A* ^*^ and *C* ^*^. Saturated regulations of nodes *B* and *C* require 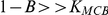, 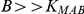, and 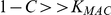, 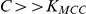, respectively. This leads to two proportional relationships, 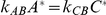, and 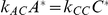. The aligned dose-response between node *A* and node *C* can be achieved by the constraint 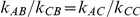. Similarly, combinational mechanisms of M3 and M5 apply to the minimal network in Figure 4d(ii). The networks in Figure 4d(iii,iv) can be viewed as combinations of the variational M1 (VM1) mechanism and the MNR mechanism (M6) where the negative self-loop of node *C* is taken over by the negative 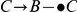 feedback loop.

### Non-minimal Functional Networks

#### Classification of robust circuits

**Figure 6:**
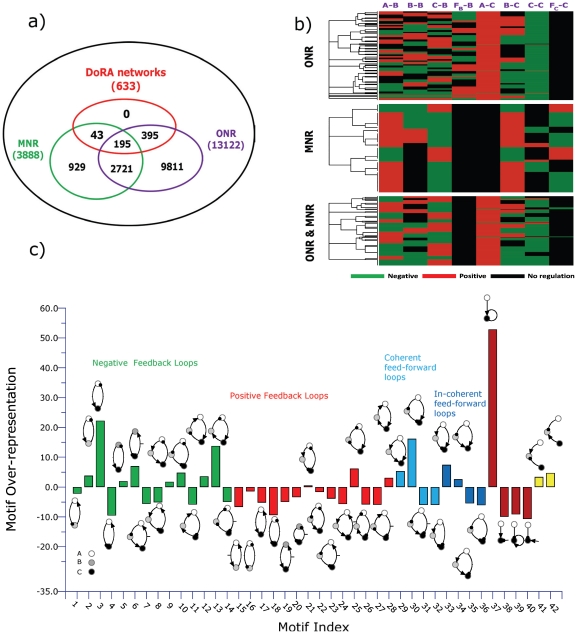
Analysis of 633 functional networks of three nodes with Q-value larger than 15. (a) Venn diagram of networks with three characters: input node directly regulates output node, input and output nodes regulate the middle buffering node with opposing signs, and both. (b) Topological clustering for DoRA networks with ONR or MNR or with both ONR and MNR. (c) Motif analysis of 633 robust DoRA networks.

The above analyses focused on minimal circuits and identified motifs that are essential for DoRA function. A minimal circuit can achieve DoRA function by a direct input-to-output link or an intermediate node that are regulated simultaneously by the input node and the output node with opposing signs. The minimal networks with DoRA function are accordingly classified fundamentally into ONR type or MNR type or ONR/MNR combinational type. But are these simple motifs and architectures the foundation for all possible DoRA networks? Are there more complex higher order solutions for DoRA that do not contain these motifs? In other words, can all possible DoRA networks be classified into ONR, MNR, and ONR/MNR categories? To address this question, we checked the topological structures of functional circuits. In the case of two-node networks, the input node always regulates directly the output node. All functional circuits contain a motif in Figure 5 (M1 to M4), and fall simply in the ONR category. To clarify structure features in the functional networks composed of three nodes, we focused on 633 DoRA circuits that have achieved DoRA function more than 15 times when 10,000 parameter sets are sampled. Analyses of these robust circuits reveal that each of them contains at least one of the motifs that are listed in Figure 5, indicating that the motifs are fundamental and necessary for DoRA function (Figure 6a,5b). In non-minimal networks, a node could be multiply activated or repressed by more than two links (e.g., Figure 4e). The kinetic equations are more complex and the scenarios for DoRA function are not so obvious as in minimal networks. Examinations of complex circuits reveal that the way for the circuits to achieve DoRA function is generally multiple but still primarily follow the mechanisms found in minimal networks. The multiplicity of ways to realize DoRA generally results in higher Q-values in these networks. A detailed kinetic analysis of the multiple ways to achieve DoRA for the specific non-minimal circuit in Figure 4e(i) is demonstrated as an example in Supporting Information S1. In general, the robust functional circuits can still be classified into ONR, MNR, and ONR/MNR categories. The Venn diagram in Figure 6a shows that, among the 633 robust circuits, there are 395 circuits of ONR type (having A-to-C link), 43 MNR type circuits (having node *B* regulated by node *A* and node *C* with opposing signs), and 195 ONR/MNR combinational type circuits (having both A-to-C link and agonistically regulated node *B* by node *A* and node *C*).

#### Clustering of functional circuits

The structure features of the robust functional circuits of three nodes can be directly perceived from the clustering of networks. The clustering of the robust circuits in each category using the Hamming distances were shown in Figure 6b, indicating the presence of common architecture features in each category. For instances, most of the ONR type circuits have a positive A-to-B link and a negative self-loop of node *C* (i.e., M1 motif is the most abundant). In these robust networks, motifs M2, M4 are almost absent (i.e., no regulation of F_C_ on node *C*), and M3 motif is scarce. The structure of MNR category is relatively simple. The pattern of A-to-B link is identical to that of B-to-C link, and is just a reverse of C-to-B link. The basal enzyme F_B_ is absent in the regulation of node *B* (i.e., there is no F_B_-to-B link), but basal enzyme F_C_ plays a role in over half of the robust MNR circuits. More profound patterns are found for the compounded category as the functional circuits share the features of both ONR and MNR circuits.

#### Overrepresented motifs

In order to explore common characters among these robust topologies of three nodes, we searched further for overrepresented motifs in these networks. Feedback loops of two nodes and three nodes as well as regulation patterns of the buffering node *B* and output node *C* are used as motifs, and their frequency of appearance in the 633 DoRA circuits was compared with ensembles of randomized networks. The results in Figure 6c reveal that prominently over-represented motifs include negative feedback loops of 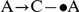 loop and 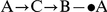
(A-activate-C-activate-B-repress-A) loop, coherent feed-forward loop 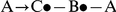 and incoherent feed-forward loop 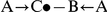. Experiments on the mating pathway in budding yeast suggested that negative feedback provide a general mechanism used in signaling systems to align dose responses and thereby increase the fidelity of information transmission [1]. The incoherent feed-forward loop was previously found over-represented for the function of adaptation [17], and was capable of the function of fold-change detection [19]. The representations of DoRA motifs in Figure 5 were depicted in Figure 6c. Motifs M1, M5, and M6 are over-represented (particularly M1), which is consistent with the observations from the clustering analysis.

## Discussion

By enumerating simple circuits for the frequently observed dynamical function of dose-response alignment in signaling systems, we have studied the organization principles for DoRA circuits. The main functional feature of DoRA circuits is to maintain a linear relationship between the input-node response and output response that is independent of the external signal. Despite the great variety of possible network architectures and various ways for achieving DoRA in interacting enzymes, our analyses suggest that the DoRA function can be resolved with a limited number of DoRA motifs. The functional motifs either consist of two nodes in which the signal is transmitted directly from the input to the output node (M1–M4), or of three nodes where the signal transduction is mediated by a buffering node that is regulated opposingly by the input and output nodes (M5, M6). The linear relationship is accomplished by dedicated constraints of the enzymatic regulations on the controlled node to approach appropriate limits (saturation or linear). This is significant because the desired linear relationships are not achieved by fine-tuning any of the network's parameters. In our analyses, Michaelis-Menten kinetics for enzymatic reactions was noncooperative (Hill coefficient = 1). When the enzymatic interactions are cooperative and are described by kinetic equations of Hill functions (Hill coefficient = n>1), the mechanisms and motifs for achieving DoRA function apply obviously also to cooperative reactions. In addition, the scenarios for achieving DoRA function should hold also for inhomogeneous cooperativity where different enzymatic regulations have different Hill coefficients. Here, we have considered only circuits of enzymatic regulations, general principles for DoRA circuits of mixed regulations of enzymes, transcription, dimerization, and degradation are also interesting and are needed in further studies.

**Figure 7:**
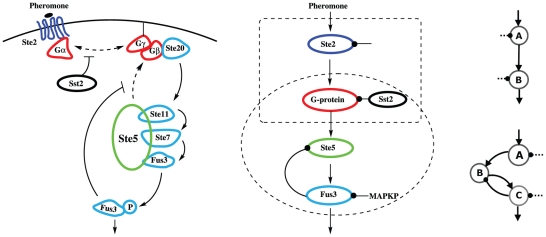
The network of mating pathway in budding yeast deduced into minimal DoRA circuits. Left: the original mating pathway in budding yeast. Middle: the simplified pathway by coarse-graining. Right: the corresponding minimal DoRA circuits of two and three nodes in Figure 4.

A well-studied biological system featured of DoRA is the pheromone response pathway in budding yeast. Experiments [1], [7], [8] and also model simulations [9] found extensive DoRAs in the pathway: the receptor binding, the G-protein activation, Ste5 membrane recruitment, and the phosphorylated Fus3pp as well as nucleus activities are all primarily well matched. After coarse-graining, we found that the mating pathway is primarily equivalent to the simple DoRA circuits that are combined in series. In the mating pathway for haploid yeast of *MAT* **a** type cells (Figure 7), the signal transduction is initiated by binding of the mating pheromone ***α***-factor to the receptor Ste2 in the plasma membrane. The receptor activates the heterotrimeric G protein that couples to it. The de-association of G protein transmits signals to multiple effectors that result in the membrane recruitment of scaffold protein Ste5 and start the mitogen-activated protein kinase (MAPK) cascade. The cascade is embedded in the scaffold protein Ste5, which consists of three kinases: Ste11 (MAPKKK), Ste7 (MAPKK), and Fus3 (MAPK). The cascade process leads finally to the phosphorylation of Fus3. Fus3p translocates into the nucleus and triggers complex changes in gene expressions for mating. In the upstream of the signaling system, the main regulator of G-protein signaling (RGS) proteins is Sst2, which increases the G-protein re-association by hydrolyzing the Gα-GTP complex and decreases downstream signal. As reported in the experiment [1], Fus3 mediates a fast negative feedback by decreasing Ste5 membrane recruitment. The negative feedback was proved to play an important role in the dose-response alignment between the receptor-pheromone binding and downstream activities. In spite that the pheromone response system consists of many detailed processes, the barebones topology of the pathway could be constructed by coarse graining. In Figure 7, the sub-units of G-protein, and the components (Ste20, Ste11, Ste7, and Fus3) that coordinate closely to form the MAPK cascade are simplified separately to a single node. Intriguingly, the simplified pheromone response network could be deduced to minimal DoRA circuits of two and three nodes (refer to Figure 4). They combine in series to form the topology that is qualitatively equivalent to the barebones of the mating pathway (Figure 7).

From the engineering perspective, biological organisms are magic designs of nature. Systems biology aims largely at unraveling design principles behind the complex systems and understanding how functions or behaviors arise out of the coordination of numerous and diversified biological components [20]. The challenge for the task is currently that one has to confront intrinsic complexities and ever-expanding databases of cellular networks. Evidences are being accumulated in recent years that diversified circuits across organisms can have followed general design principles [19], [21]–[25]. Transcriptional regulatory networks were found to make virtually of a small set of recurring network motifs [21], [24], [26]. Simple network topologies associated with specific dynamical functions were found for reliable all-or-none switches and bistability [27], [28], design principles of biochemical oscillators [29], adaptations [17] and fold-change detections [19], [30]. The design and mechanism of band-pass filter for time-limited oscillations which is physiologically significant for selective regulation of cellular processes were also reported [31], [32]. While the dynamical function of DoRA has been well documented and commonly observed in many signaling systems, its implication, general consequences, and design principles have been seldom investigated [1]. The function-motif recipes and simple circuits for DoRA reported here represent virtually the guiding principle for designing DoRA networks, and hopefully help us to understand DoRA signaling systems across organisms. It would be fascinating to test whether natural DoRA signaling systems could be well resolved on the base of the simple DoRA circuits.

## Methods

### Screening for DoRA networks

We limited ourselves to enzymatic regulatory networks and modeled network links using Michaelis-Menten kinetics. There are totally 54 two-node and 16038 three-node networks to be investigated. For each particular circuit architecture, we sampled 10,000 sets of parameters for the Michaelis-Menten equations uniformly in logarithmic scale in the parameter space using the Latin hypercube sampling method [17], [18]. The stationary solutions of the ordinary differential equations were obtained by numerical integration of the equations with the fourth order Runge Kutta method. The solutions were examined in parallel by numerical solving of the corresponding stationary nonlinear algebra equations. The dose-response curves for each set of parameter were then obtained as the input level is tuned between [0,1]. The dose-response alignment was considered to be achieved in case the linear correlation coefficient between the output node and the input node is larger than 0.99. We defined the Q-value for each particular circuit architecture as the number of parameter sets out of 10,000 parameter samples with which the DoRA function is achieved.

### Clustering of functional networks

In three-node networks, the DoRA function is controlled by node *B* and (or) node *C*, we considered eight possible links (as each node has four possible links from nodes *A*, *B*, *C* and basal enzymes F_A_, F_B_, F_C_) of node *B* and node *C* for a circuit. Each link was assigned with a value of 1, −1, or 0 (1 for positive regulation, −1 for negative regulation, and 0 for no regulation). A circuit was thus represented by a sequence of length eight. Hamming pair-wise distance was defined for two networks as the number of regulations that differ in the two networks. The clustering property is then calculated from the distance matrix using the function *clustergram* in the software Matlab.

### Over-represented Motif

The calculation of motif overrepresentation was performed by randomizing the 633 DoRA functional networks: select two networks at random and exchange their links at a randomly selected position (such as *A*-to-*B*) when and only when there is a link (but different) in both networks at this position. We generated 1000 randomized ensembles of 633 networks in this way. For a specific motif, the mean number of appearance 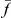 and the standard deviation *d* of this motif in these ensembles are then calculated. Using the number of appearance of the motif in the original 633 DoRA networks *f*, the overrepresentation of the motif is calculated as 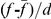.

## Supporting Information

Supporting Information S1 Mathematical analysis of a non-minimal circuit.
(DOC)
