# Supplementary material for: Dose-Response Aligned Circuits in Signaling Systems
Source: PLoS One. 2012 Apr 5;7(4):e34727. doi: 10.1371/journal.pone.0034727 (PMC3320644; doi:10.1371/journal.pone.0034727)
Supplement: Supporting Information S1 — Mathematical analysis of a non-minimal circuit. (DOC) [file pone.0034727.s001.doc]

**Supporting Information**

**Mathematical analysis of a non-minimal circuit -** In non-minimal networks, the nodes would be regulated in complex ways, i.e., a node could be multiply activated or repressed by more than two links (Figure 4e). The kinetic equations become complex and the scenarios for DoRA function are not as obvious as in the minimal networks. Examinations of complex circuits reveal that the way for the circuits to achieve DoRA function is generally multiple. This is the reason why more complex topologies often have higher Q-values than the minimal circuits. In the following, we check into the network of Figure 4e(i) which has a relatively high Q-value as a specific example. The network, in which node *B* and node *C* have duple deactivation regulations, is described by the following kinetic equations,

$\left\{\begin{matrix} \frac{dA}{dt}=\frac{k_{I}I(1-A)}{1-A+K_{MI}}-\frac{k_{CA}CA}{A+K_{MCA}}, \\ \frac{dB}{dt}=\frac{k_{CB}C(1-B)}{1-B+K_{MCB}}-\frac{k_{AB}AB}{B+K_{MAB}}-\frac{k_{BB}B^{2}}{B+K_{MBB}}, \\ \frac{dC}{dt}=\frac{k_{AC}A(1-C)}{1-C+K_{MAC}}-\frac{k_{BC}BC}{C+K_{MBC}}-\frac{k_{CC}C^{2}}{C+K_{MCC}}. \end{matrix}\right.$ (s1)

Mathematically, the circuit has several ways to achieve DoRA function. Firstly, the DoRA function can be achieved through the intermediate node *B*, *i.e.*, the MNR mechanism. If the repression of node *A* and activation of node *C* on node *B* work in saturation region, we have $K_{MAB}<<B$,$K_{MCB}<<1-B$. In addition, if the Michaelis-Menten constant $K_{MBB}$ is constrained as $K_{MBB}>>1$, we have a trivial term $k_{BB}BB/(B+K_{MBB})$ which could be dropped. The second equation of (s1) is thus reduced to the obvious DoRA form, $dB/dt=k_{CB}C-k_{AB}A$. Secondly, the circuit can alternatively achieve DoRA by regulating the active form of enzyme *B* at a constant level. From equation (s1), when the concentration *B* is fixed as independent on the input level *I*, it is obvious that the stationary state of the second equation exhibits a linear relation between the concentrations *A^*^* and *C^*^*,

$C^{*}=\frac{(1-B^{*}+K_{MCB})k_{AB}B^{*}}{k_{CB}(1-B^{*})(B^{*}+K_{MAB})}A^{*}+\frac{(1-B^{*}+K_{MCB})k_{BB}B^{*2}}{k_{CB}(1-B^{*})(B^{*}+K_{MBB})}$. (s2)

The mechanism for the concentration *B* to be fixed as constant and be independent on the input level *I* is identical to the one that gives rise to adaptation. Adaptation is characterized as the system’s ability to respond to a change in the input stimulus and recover the pre-stimulated output level. When node *B* is appointed as the output node instead of node *C*, the circuit of Figure 4e(i) is an incoherent feed-forward loop with a proportional node that has additional negative feedback loops. The mechanism that gives rise to adaptation in this kind of circuits has been discussed in detail [10]. Examinations of the parameter sets reveal that the third way the circuit achieves DoRA is by over-repressing node *B* so that the active form of enzyme *B* is at a very low level. By further constraining $K_{MBB}>>B$, one has $k_{BB}B^{2}/(B+K_{MBB})\approx0$ as $B\approx0$. The linear relationship between stationary concentrations *A*^*^ and *C*^*^ is established from the second equation of (s1) by constraining both the repression of node *A* and activation of node *C* on node *B* to saturation, *i.e.*, $K_{MAB}<<B$,$K_{MCB}<<1-B$.

The circuit can alternatively achieve the DoRA function through the output node *C*, i.e., the ONR mechanism. Firstly, if the activation of node *A* on node *C* and the negative self-loop of node *C* are constrained to saturation, we have $K_{MAC}<<1-C$ and $K_{MCC}<<C$. The third equation of (s1) is reduced to the DoRA form$dC/dt=k_{AC}A-k_{CC}C$ by limiting $k_{BC}/K_{MBC}<<1$ which guarantees the approximation $k_{BC}BC/(C+K_{MBC})\approx0$. Secondly, the circuit can establish the linear relationship between *A*^*^ and *C*^*^ by regulating coordinately both node *B* and node *C*. The linear relationship between stationary concentrations *B*^*^ and *C*^*^ could be first achieved by limiting the parameters as $K_{MCB}<<1-B$, $K_{MBB}<<B$, and $K_{MAB}>>k_{AB}$. Meanwhile, when $K_{MAB}$ is much larger than 1.0, the term $k_{AB}AB/(B+K_{MAB})$ in the equation for node *B* could be neglected. The second equation of (s1) is thus simplified as,

$dB/dt=k_{CB}C-k_{BB}B$. (s3)

When the regulations on node *C* are constrained to saturation, i.e., $K_{MAC}<<1-C$, $K_{MCC}<<C$, and $K_{MBC}<<C$, the third equation of (s1) can be simply written as,

$dC/dt=k_{AC}A-k_{BC}B-k_{CC}C$. (s4)

At stationary state, the linear relationship between *A*^*^ and *C*^*^ are obvious from equations (s3) and (s4).
